# Finding the closed-form solutions of dissipative oscillatory systems

**DOI:** 10.1038/s41598-022-08418-3

**Published:** 2022-03-22

**Authors:** Saba Irum, Imran Naeem

**Affiliations:** Department of Mathematics, Lahore University of Management Sciences, Lahore, Pakistan

**Keywords:** Applied mathematics, Computational science

## Abstract

This paper shows how to use the approximate Hamiltonian approach for the non-conservative system not capable of possessing Hamiltonian. Using the approximate Hamiltonian method for a non-conservative system is not possible in general. We propose a way to obtain the closed-form solutions for such systems. We use the approximate dual Hamiltonian method to construct the first integrals and closed-form solutions of the Van der Pol equation. First the solutions of the initial value VdP equation is obtained using approximate dual Hamiltonian method. Then a good agreement is observed in the comparison between the numerical results and the results through approximate dual Hamiltonian method. Finally, we use the approximate dual Hamiltonian method to find the dual Hamiltonian and first integrals of the forced Van der Pol oscillator and Liénard system. These significant results can be applied to any Van der Pol equation.

## Introduction

The van der pol equation is a classical standard in chaos theory. The researchers used this model in biological and physical sciences from early development stages. This equation is considered as an example of an oscillator with nonlinear damping. Several authors and researchers attempted the search for an analytical solution to the van der pol equation. Van der pol^1^ was the first who modeled the electrical circuits in a vacuum. The equation is often studied for its rich set of chaotic solutions^2^, and as a prototype for testing numerical schemes. There is no existing general theory for deriving a nonlinear differential equation’s closed-form solutions. Therefore more and more attention was concentrated on numerical methods to obtain solutions (some of them are listed in^3^) and on approximated analytic ways based on either linearization^4^ or perturbative approach or adomian decomposition^5^. Some methods to this end are Lie, and related symmetry methods^6^. To analyze tha Oscillatory behavior the parameter plays an important role. There are different methods to calculate these parameters^7^. Among these methods, the Baikov^8–10^ provided the approximate version of the Lie theorem. Naeem and Mahomed^11,12^ developed the partial Lagrangian approach^13,14^ for the perturbed ODEs. A lot of studies have been established for the approximate Hamiltonian systems. Different perturbation techniques have been developed to establish the approximate first integral, e.g., direct method^15^, Brikhof-Gustavson normal form method^16^, and other methods are described in^17^. Naz and Naeem^18^ developed the approximate partial Noether approach to construct approximate first integrals for the approximate partial Hamiltonian systems. They also provided the approximate Noether symmetry determining equation for the approximate first integrals of the approximate Hamiltonian system^19^.

Moreover, these methods have a limited scope due to the absence of Hamiltonian in the dissipative systems. This study shows that an improvised version of Batman’s dual Hamiltonian for DSHO can be used for such nonlinear systems. Batman’s dual Hamiltonian yields one usual equation for DSHO along with one auxiliary equation. Shah^20^ obtained a perturbative technique for non-conservative systems. Our aim is to introduce a Hamiltonian formalism for non-conservative systems. Thus we have developed an approximate dual Hamiltonian method for dissipative systems. The motivation of this study was the apparent failure to write the Hamiltonian for the non-conserved system.

In order to see the effectiveness of the approach, we have chosen the Van der Pol oscillator (VdPO), a widely studied non-conservative system. One can easily extend the results obtained for VdPO to other non-conservative systems. In this method, the approximate Hamiltonian approach has been utilized to find the closed-form solutions of the non-conservative system. The above statement appears contrary because the approximate Hamiltonian approach is explicitly defined for the Hamiltonian system. Once the approximate Hamiltonian of the system is formalized, we can easily calculate the closed-form solutions. Developing such a method must be appreciated because one can find closed-form solutions for the non-conservative system. The approach of bifurcation analysis is widely utilized to examine attractors and their influence on the dynamics of the system. Even though this is not our main interest, it is fascinating to investigate it as well in the future.

This paper utilizes the Hamiltonian description for the DSHO to construct the Hamiltonian of VdPO and other non-conservative systems. We illustrate how our method can yield the approximate first integrals and solutions. We discuss the approximate dual Hamiltonian of the VdPO in "Hamiltonian for conserved van der pol oscillator" section and then proceed to construct the approximate first integrals and closed-form solutions in "Closed form solutions of van der pol oscillator" section. In "Closed form solutions of van der pol oscillator" section, we present a novel application of ADHM to VDP initial value problems and compares the results with the numerical results. Before concluding in "Conclusions" section, we computed the approximate dual Hamiltonian of the forced Van der Pol equation and Liénard equation in "Forced van der pol oscillator" and "Liénard system" sections.

## Hamiltonian for conserved van der pol oscillator

The two-dimensional dynamical system for the Van der Pol oscillator possesses well-known results in the literature.

1 $$\begin{aligned} \ddot{x}+\varepsilon (\alpha x^2+\beta y^2-1){\dot{x}}+\omega ^2 x=0. \end{aligned}$$

The oscillatory behavior of the VdP Eq. (1) is effected by the damping force $(1-\alpha x^2-\beta y^2){\dot{x}}$, where $x(t),\ y(t)$ are displacement.

When considering the phase-space coordinates, the presence of attractors makes it impossible to construct the Hamiltonian for this autonomous dynamical system. Since the search for the Hamiltonian is a difficult task, adopting a strategy of introducing an auxiliary system that complements the Van der Pol oscillator is a good option to proceed with the construction of the Hamiltonian. We make the same choice and add an auxiliary equation to construct the Hamiltonian. It is worth noting here that the auxiliary Van der Pol oscillator does not affect the dynamics of the Van der Pol oscillator. The four dimensional Van der Pol oscillator system is defined by the following second order nonlinear differential equations

2 $$\begin{aligned} \ddot{x}+\varepsilon (\alpha x^2+\beta y^2-1){\dot{x}}+\omega ^2 x=0,\nonumber \\ \ddot{y}-\varepsilon (\alpha x^2+\beta y^2-1){\dot{y}}+\omega ^2 y=0, \end{aligned}$$

where $\alpha ,\ \beta$ are real parameters. In general, $\varepsilon$ is a parameter that indicates the damping strength and non-linearity. We assume very small damping, which means $\varepsilon$ is a very small parameter. The addition of the auxiliary nonlinear two-dimensional system complementing the ordinary Van der Pol oscillator so that the total system becomes conservative. Since the dynamics of the well known Van der Pol oscillator are not affected by the coupling. This leads to establishing a way for defining Hamiltonian using Legendre transformation.

Many authors analyze the Van der Pol oscillator using complex coordinates. The use of complex coordinates transformed the system, and with this change of variable, a Hamiltonian was associated. Putting $\beta = 0$, we get the Van der Pol equation in a single variable, *x*‘ only, and this can be transformed easily into a complex variable equation. But when $\beta \ne 0$, the complex variable equation is not easily solvable. So we choose the dual Hamiltonian method, which is more straightforward. One can note that due to the perturbed nature of the system, the best way is to define an approximate Lagrangian for the system as

3 $$\begin{aligned}&L(t,x,y, {\dot{x}}, {\dot{y}})={\dot{x}}{\dot{y}}-\omega ^2 x y +\frac{\varepsilon }{2}\Bigg [\Bigg(y-\alpha y x^2 -\frac{\beta y^3}{3}\Bigg){\dot{x}}-\Bigg(x-\beta x y^2 -\frac{\alpha x^3}{3}\Bigg){\dot{y}}\Bigg ]. \end{aligned}$$

Using the approximate Legendre transformation, the approximate Hamiltonian is found to be

4 $$\begin{aligned}&H(t,x,y,{\dot{x}},{\dot{y}})=p_x {\dot{x}}+p_y {\dot{y}}-L, \end{aligned}$$

5 $$\begin{aligned} \text {where}\quad&p_x=\frac{\partial L}{\partial {\dot{x}}}= {\dot{y}}+\varepsilon \Bigg(\frac{y-\alpha y x^2-\frac{\beta y^3}{3} }{2} \Bigg),\end{aligned}$$

6 $$\begin{aligned}&p_y=\frac{\partial L}{\partial {\dot{y}}}={\dot{x}}-\varepsilon \Bigg(\frac{x-\beta x y^2-\frac{\alpha x^3}{3}}{2}\Bigg). \end{aligned}$$

Finally, from (3), (5) and (6)

7 $$\begin{aligned} H=p_x p_y+\omega ^2 x y +\frac{\varepsilon }{2}\Bigg [\Bigg(x-\beta x y^2 -\frac{\alpha x^3}{3}\Bigg)p_x-\Bigg(y-\alpha y x^2 -\frac{\beta y^3}{3}\Bigg)p_y\Bigg ]. \end{aligned}$$

The Hamiltonian is written as a combination of an unperturbed and perturbed Hamiltonian:

$$\begin{aligned} H=H_0+\varepsilon H_1, \end{aligned}$$

where $H_0=p_x p_y+\omega ^2 x y$ is unperturbed Hamiltonian and $H_1=\frac{1}{2}\Bigg [(x-\beta x y^2 -\frac{\alpha x^3}{3})p_x-(y-\alpha y x^2 -\frac{\beta y^3}{3})p_y\Bigg ]$ is the perturbed Hamiltonian of first order.

## Closed form solutions of van der pol oscillator

The approximate Hamiltonian (7) is used to construct the following approximate Hamiltonian system

8 $$\begin{aligned}&{\dot{x}}=p_y+\frac{\varepsilon }{2}(x-\beta x y^2-\frac{\alpha x^3}{3})\ ,\nonumber \\&\dot{p_x}=-\frac{\partial H}{\partial {\dot{x}}}\ ,\nonumber \\&{\dot{y}}=p_x-\frac{\varepsilon }{2}(y-\alpha y x^2 -\frac{\beta y^3}{3})\ ,\nonumber \\&\dot{p_y}=-\frac{\partial H}{\partial {\dot{y}}}\ . \end{aligned}$$

The approximate Hamiltonian (7) and approximate Hamiltonian system (8) give rise to the following approximate Noether symmetries determining equation

9 $$\begin{aligned}&p_x(\eta ^1_{0t}+\varepsilon \eta ^1_{1t}+(\eta ^1_{0x}+\varepsilon \eta ^1_{1x})(p_y+\frac{\varepsilon }{2}(x-\beta y^2 x -\frac{1}{3} \alpha x^3))+(\eta ^1_{0y}+\varepsilon \eta ^1_{1y})\nonumber \\&(p_x-\frac{\varepsilon }{2}(y-\alpha y x^2 -\frac{1}{3} \beta y^3)))+p_y(\eta ^2_{0t}+\varepsilon \eta ^2_{1t}+(\eta ^2_{0x}+\varepsilon \eta ^2_{1x})\nonumber \\&(p_y+\frac{\varepsilon }{2}(x-\beta y^2 x -\frac{1}{3} \alpha x^3))+(\eta ^2_{0y}+\varepsilon \eta ^2_{1y})(p_x-\frac{\varepsilon }{2}(y-\alpha y x^2 -\frac{1}{3} \beta y^3)))- \nonumber \\&(\eta ^1_0+\varepsilon \eta ^1_1)(\omega ^2 y+\frac{\varepsilon }{2}(-\alpha x^2 -\beta y^2+1)p_x+2\alpha x y p_y)-(\eta ^2_0+\varepsilon \eta ^2_1)\nonumber \\&(\omega ^2 x+\frac{\varepsilon }{2}(-2\beta x y p_x -(-\alpha x^2-\beta y^2+1)p_y)-(\xi _{0t}+\varepsilon \xi _{1t}+(\xi _{0x}+\varepsilon \xi _{1x})\nonumber \\&(p_y+\frac{\varepsilon }{2}(x-\beta y^2 x -\frac{1}{3} \alpha x^3))+(\xi _{0y}+\varepsilon \xi _{1y})(p_x-\frac{\varepsilon }{2}(y-\alpha y x^2 -\frac{1}{3} \beta y^3)))\nonumber \\&(p_x p_y+\omega ^2 x y +\frac{\varepsilon }{2}((x-\beta x y^2 -\frac{\alpha x^3}{3})p_x-(y-\alpha y x^2 -\frac{\beta y^3}{3})p_y))\nonumber \\&=B_{0t}+\varepsilon B_{1t}+(B_{0x}+\varepsilon B_{1x})(p_y+\frac{\varepsilon }{2}(x-\beta y^2 x -\frac{1}{3} \alpha x^3))\nonumber \\&+(B_{0y}+\varepsilon B_{1y})(p_x-\frac{\varepsilon }{2}(y-\alpha y x^2 -\frac{1}{3} \beta y^3)), \end{aligned}$$

where we used $\xi (t,x,y)=\xi _0(t,x,y)+\varepsilon \xi _1(t,x,y), \ \eta ^1(t,x,y)=\eta ^1_{0}(t,x,y)+\varepsilon \eta ^1_{1}(t,x,y), \ \eta ^2(t,x,y)=\eta ^2_{0}(t,x,y)+\varepsilon \eta ^2_{1}(t,x,y) \ \text {and}\ B(t,x,y)=B_{0}+\varepsilon B_{1}$. To find the zeroth order and first order approximations, we separate (9) with respect to $\varepsilon ^0$ and $\varepsilon ^1$ respectively. We obtain the following system of equation for zero order approximation:

Zeroth order approximation:

10 $$\begin{aligned} \xi _{0x}&=0,\nonumber \\ \eta ^2_{0x}&=0,\nonumber \\ \eta ^1_{0y}&=0,\nonumber \\ \eta ^1_{0x}+\eta ^2_{0y}-\xi _{0y}-\xi _{0t}&=0,\nonumber \\ B_{0x}+\eta ^2_{0t}&=0,\nonumber \\ \xi _{0y} \omega ^2 x y-\eta ^1_{0t}+B_{0y}&=0,\nonumber \\ \omega ^2(-\xi _{0t} x y-\eta ^1_{0} y -\eta ^2_{0} x) -B_{0t}&=0. \end{aligned}$$

After some calculations, the first order approximation yields the following system:

11 $$\begin{aligned}&\xi _{1y}=0,\nonumber \\&\eta ^2_{1x}=0,\nonumber \\&\xi _{0y}\left( \frac{1}{2}x-\frac{1}{2}\beta x y^2 -\frac{1}{6}\alpha x^3\right) +\eta ^1_{1y}=0,\nonumber \\&2 \xi _{0y}\left( \frac{1}{2}y-\frac{1}{2}\alpha y x^2 -\frac{1}{6}\beta y^3\right) +\xi _{1t}-\eta ^2_{1y}-\eta ^1_{1x}=0,\nonumber \\&\eta ^2_{0} \beta x y -B_{1y}-(\eta ^1_{0}+\xi _{0t} x+ \xi _{0y}y)\left( -\frac{1}{2} \alpha x^2-\frac{1}{2}\beta y^2 +\frac{1}{2}\right) =0,\nonumber \\&(\eta ^2_{0}+\xi _{0t} y)\left( \frac{1}{2}\alpha x^2+\frac{1}{2}\beta y^2 -\frac{1}{2}\right) +\eta ^1 \alpha x y-\eta ^2_{1t}+B_{1x}=0,\nonumber \\&(B_{0y}-\xi _{0y}\omega ^2 x y)\left( -\frac{1}{2}y+\frac{1}{2}\alpha y x^2 +\frac{1}{6}\beta y^3\right) +\omega ^2(\eta ^1_{1} y+\eta ^2_{1} x-\xi _{1t} x y)+\nonumber \\ {}&(B_{0x}-\xi _{0x}\omega ^2 x y)\left( \frac{1}{2}x-\frac{1}{2}\beta x y^2 -\frac{1}{6}\alpha x^3\right) +B_{1t}=0. \end{aligned}$$

In the search for the solution of system of Eq. (11) with the aid of (10) we arrive at different combinations for the parametric values $\alpha$ and $\beta$. We discuss the approximate Noether symmetry operator and approximate first integrals for each possible values of $\alpha$ and $\beta$. Note that these cases arise automatically in the solution procedure.

*Case* 1 $$\alpha =0, \ \beta =0.$$

For $\alpha =0, \ \beta =0$, we obtain the following eight stable and seven unstable approximate first integrals associated with the approximate Noether symmetries.

12 $$\begin{aligned} I_1&=p_x x-p_y y,\nonumber \\ I_2=&\, \omega \cos (2 \omega t)(p_x x+p_y y)-\sin (2 \omega t)(p_x p_y-\omega ^2 x y+\frac{1}{2}\varepsilon (p_x x-p_y y))\nonumber \\&+\,\varepsilon \Bigg [ p_x \sin (2 \omega t)x+\frac{1}{2\omega }\cos (2\omega t)(p_x p_y+\omega ^2 xy)-\cos (2\omega t)\omega xy\Bigg ]+O(\varepsilon ^2),\nonumber \\ I_3=&-\, \omega \sin (2 \omega t)(p_x x+p_y y)-\cos (2 \omega t)(p_x p_y-\omega ^2 x y+\frac{1}{2}\varepsilon (p_x x-p_y y))\nonumber \\&+\,\varepsilon \Bigg [ p_x \cos (2 \omega t)x-\frac{1}{2\omega }\sin (2\omega t)(p_x p_y+\omega ^2 xy)+\sin (2\omega t)\omega xy\Bigg ]+O(\varepsilon ^2),\nonumber \\ I_4&=-p_x p_y-\omega ^2 x y-\frac{1}{2}\varepsilon \Bigg [x p_x-y p_y\Bigg ],\nonumber \\ I_5&=p_y\sin (\omega t)-\cos (\omega t)\omega x-\frac{1}{2 \omega }\varepsilon p_y\cos (\omega t),\nonumber \\ I_6&=p_y\cos (\omega t)+\sin (\omega t)\omega x+\frac{1}{2 \omega }\varepsilon p_y\sin (\omega t),\nonumber \\ I_7&=p_x\sin (\omega t)- \omega y \cos (\omega t)-\frac{1}{2}\varepsilon \Bigg [-\frac{p_x}{ \omega }(\cos (\omega t)+\omega t \sin (\omega t))-\sin (\omega t)y+\omega t \cos (\omega t)y\Bigg ],\nonumber \\ I_8&=p_x\cos (\omega t)+ \omega y \sin (\omega t)+\frac{1}{2}\varepsilon \Bigg [p_x\cos (\omega t)t+\omega t \sin (\omega t)y\Bigg ],\nonumber \\ I_{9}&=-\varepsilon \Bigg [p_x p_y+\omega ^2 x y\Bigg ]+O(\varepsilon ^2),\nonumber \\ I_{10}&=\varepsilon \Bigg [\frac{1}{2}p_x x-\frac{1}{2} p_y y\Bigg ],\nonumber \\ I_{11}&=\varepsilon \Bigg [\cos (2 \omega t)(p_x x+p_y y)+(\omega ^2 xy-p_x p_y) \frac{sin(2 \omega t)}{\omega }\Bigg ]+O(\varepsilon ^2),\nonumber \\ I_{12}&=\varepsilon \Bigg [(x p_x+y p_y)\sin (2\omega t)+\frac{ \cos (2\omega t)}{\omega }(- \omega ^2 xy+p_x p_y)\Bigg ]+O(\varepsilon ^2),\nonumber \\ I_{13}&=-2\varepsilon \Bigg [p_x p_y+\omega ^2 x y\Bigg ]+O(\varepsilon ^2),\nonumber \\ I_{14}&=\varepsilon \bigg [p_x \sin (\omega t)-\omega y \cos (\omega t)\Bigg ],\nonumber \\ I_{15}&=\varepsilon \bigg [p_x \cos (\omega t)+\omega y \sin (\omega t)\Bigg ]. \end{aligned}$$

The next step is to compute the solutions of Eq. (2) using the approximate first integrals discussed in Eq (12). It is worth mentioning that each first integral can be written as a specific constant and can be determined by the given initial/boundary condition.

The values of *x* and *y* are obtained in Eq. (13) by using $I_1=c_1,\ I_4=c_4 ,\ I_5=c_5$ and $I_6=c_6$ .

13 $$\begin{aligned} x( t )&=\,{\frac{-\sin ( \omega \,t ) { c_{5}}\,\varepsilon +2\,\sin ( \omega \,t ) { c_{6}}\,\omega -2\, \cos ( \omega \,t ) { c_{5}}\,\omega -\cos ( \omega \,t ) { c_{6}}\,\varepsilon }{{2 \omega }^{2}}},\nonumber \\ y( t )&=-{\frac{ \cos ( \omega \,t ) (A\,c_{6}-B\, c_{5} \omega ) +\sin ( \omega \,t ) (A\,c_{5}-B\, c_{6} \omega ) }{ ( {{ c_{5}}}^{2}+4\,{{ c_{6}}}^{2} ) {\omega } ^{2}+{ c_{5}}\,{ c_{6}}\,\omega \,\varepsilon + ( ( {{ c_{5} }}^{2}-{{ c_{6}}}^{2} ) \omega ) \varepsilon \,\sin ( \omega \,t ) \cos ( \omega \,t ) }},\nonumber \\ \text {where}\ A&= 4 c_{1}\,{\omega }^{2}-\varepsilon \, { c_{1}}\,\varepsilon -2\,\varepsilon \,{ c_{4}}, B=- {2 c_{1}}\,\varepsilon -4\,{ c_{4}}. \end{aligned}$$

Similarly, using different combinations of first integrals give rise to

14 $$\begin{aligned} x( t )&={\frac{2\,\sin ( \omega \,t ) { c_{1}}\,\omega \,\varepsilon +2\,\sin ( \omega \,t ) { c_{5}}\,{ c_{14}}-2\,\cos ( \omega \,t ) { c_{5}}\,{ c_{15}}}{-\sin ( \omega \,t ) \cos ( \omega \,t ) { c_{15}}\,\varepsilon - ( \cos ( \omega \,t ) ) ^{2}{ c_{14}}\,\varepsilon +2\,{ c_{15}}\,\omega }},\nonumber \\ y( t )&={\frac{\sin ( \omega \,t ) { c_{14}}-{ c_{15}}\,\cos ( \omega \,t ) }{\omega \,\varepsilon }}, \end{aligned}$$

and

15 $$\begin{aligned} x( t )&={\frac{-\sin ( \omega \,t ) ( { c_{5}}\,\varepsilon -2\,{ c_{6}}\,\omega ) -\cos ( \omega \,t ) ( 2\,{ c_{5}}\,\omega +{ c_{6}}\,\varepsilon ) }{2{\omega }^{2}}},\nonumber \\ y( t )&={\frac{\sin ( \omega \,t )(-2\, { c_{1}}\,{\omega }^{2}\varepsilon - { c_{5}}\,{ c_{15}}\,\varepsilon +2\, { c_{6}}\,{c_{15}}\,\omega )-\cos ( \omega \,t ) { c_{5}}\,{ c_{15}}\, ( 2\,\omega +\varepsilon ) }{2{ c_{5}}\,{\omega }^{2}\varepsilon }}, \end{aligned}$$

16 $$\begin{aligned} x( t )&={\frac{2\,\cos ( \omega \,t ) { c_{1}}\,\omega \,\varepsilon +2\,\sin ( \omega \,t ) { c_{6}}\,{ c_{14}}-2\,\cos ( \omega \,t ) { c_{6}}\,{ c_{15}}}{2\,{ c_{14}}\,\omega + ( \sin ( \omega \,t ) )^{2}{ c_{15}}\,\varepsilon +\sin ( \omega \,t ) \cos ( \omega \,t ) { c_{14}}\,\varepsilon }},\nonumber \\ y( t )&={\frac{\sin ( \omega \,t ) { c_{14}}-{ c_{15}}\,\cos ( \omega \,t ) }{\varepsilon \,\omega }}, \end{aligned}$$

17 $$\begin{aligned} x(t)&=\frac{\sin(\omega \, t)\, C + \cos(\omega \, t) \, D}{c_{14}^2 \omega+c_{15}^2 \omega}, \nonumber \\ y(t) &=\frac{\sin (\omega \, t) c_{14}-\cos(\omega\, t) c_{15}}{\epsilon \, \omega},\\ \text{where} \, C&=-c_{10}\, c_{14}\, \epsilon +2 \, c_{10} \, c_{15} \, \omega +c_{9}\, c_{14}, \nonumber \\ D&=2 c_{10}\, c_{14}\, \omega +c_{10} c_{15}\, \epsilon +c_{9}\, c_{15}. \nonumber\end{aligned}$$

18 $$\begin{aligned} x( t )&={\frac{-\sin ( \omega \,t ) E -\cos ( \omega \,t ) F}{(2\, \sin ^{2}( \omega \,t ) { c_{14}} -\sin ( \omega \,t ) \cos ( \omega \,t ) { c_{15}} + \cos ^{2}( \omega \,t ) { c_{14}})\,\omega \,\varepsilon -2\,{ c_{15}}\,{\omega }^{2} }}, \nonumber \\ y( t )&={\frac{\sin ( \omega \,t ) { c_{14}}-{ c_{15}}\,\cos ( \omega \,t ) }{\varepsilon \,\omega }}, \nonumber \\ \text {where} \,E&=( -{ c_{6}}\,{ c_{14}}\,\varepsilon +2\,{ c_{6}}\,{ c_{15}}\,\omega +{ c_{9}}\,\varepsilon ),\quad F=( 2\,{ c_{6}}\,{ c_{14}}\,\omega +{ c_{6}}\,{ c_{15}}\,\varepsilon +2\,{ c_{9}}\,\omega ) . \end{aligned}$$

We can construct different solutions by utilizing various combinations of the first integrals. We don’t include all of them in this study, but only some of the results are discussed here. When $\alpha =\beta =0,$ the given system represents a linear oscillator, and the fundamental behavior of this type of oscillator is also captured in the proposed method. The solution of linear oscillator is $x(t)=\mathrm {e}^{\frac{\varepsilon t}{2}}\cos (\omega _0 t+\phi )$ which is obtained by some manipulations in (19).

19 $$\begin{aligned} x( t )&={\frac{-\sin (\omega _0\ t ){ c_{5}}\ \varepsilon + \cos ( \omega _0\ t) { c_{6}}\ \varepsilon }{{2 \omega }^{2}}} =\mathrm {e}^{\frac{ \varepsilon t}{2}} \cos (\omega _0 t+\phi ),\nonumber \\ y( t )&={\frac{-\sin (\omega _0\ t ){ c_{5}}\ \varepsilon +2\ \sin ( \omega _0\ t ) { c_{6}}\ \omega -2\ \cos ( \omega _0\ t ) { c_{5}}\ \omega -\cos ( \omega _0\ t) { c_{6}}\ \varepsilon }{{2 \omega }^{2}}}, \end{aligned}$$

where $$\omega _0=\frac{\sqrt{\varepsilon ^2-4 \omega }}{2}.$$

*Case* 2 $$\alpha =0, \ \beta \ne 0.$$

When $\alpha =0, \ \beta \ne 0$, the ADHM yields five repeated approximate first integrals $I_{4},I_{9},\ I_{11},\ I_{12},\ I_{13}$ along with four additional approximate first integral $I_{16},\ I_{17}, \ I_{18},\ I_{19}$.

$$\begin{aligned} I_{16}&=p_x p_y+\omega ^2 x y+\frac{1}{2}\varepsilon \Bigg [(-\beta x y^2+x) p_x-(y-\frac{1}{3}\beta y^3)p_y\Bigg ],\\ I_{17}&=-p_x p_y-\omega ^2 x y-\frac{1}{2}\varepsilon \Bigg [p_y y-p_x x+(-\beta x y^2+x) p_x-(y-\frac{1}{3}\beta y^3)p_y\Bigg ],\\ I_{18}&=-1+\varepsilon \Bigg [p_x\cos (\omega t)+\omega y\sin (\omega t)\Bigg ],\\ I_{19}&=\varepsilon \Bigg [p_x \sin (\omega t)-\cos (\omega t)\omega y-\omega ^2 xy-p_x p_y\Bigg ]+O(\varepsilon ^2). \end{aligned}$$

Now, we utilize these approximate first integrals to derive the solutions. Using $I_4=c_4,\ I_9=c_9,\ I_{17}=c_{17}$ and $I_{18}=c_{18}$ and solving the resulting equations yield

20 $$\begin{aligned} x ( t )&=-{\frac{{ c_{9}}}{\sin ( \omega \,t ) \omega ( { c_{18}}+1 ) -\cos ( \omega \,t ) \omega \, ( { c_{17}}-{ c_{9}} ) }},\nonumber \\ y ( t )&={\frac{\sin ( \omega \,t ) ( {c_{18}}+1 ) +\cos ( \omega \,t ) ( { c_{9}}-{ c_{17}} ) }{\varepsilon \,\omega }}, \end{aligned}$$

21 $$\begin{aligned} x ( t )&={\frac{ \cos ( \omega \,t ) G - \sin ( \omega \,t) K }{\omega \,\varepsilon \, ( {{ c_{9}}}^{2}-2\,{ c_{9}}\,{ c_{17}}+{{ c_{18}}}^{2}+{{ c_{17}}}^{2}+2\,{ c_{18}}+1 ) }},\nonumber \\ y ( t )&={\frac{\cos ( \omega \,t ) ( { c_{9}}-{ c_{17}} ) +\sin ( \omega \,t ) ( { c_{18}}+1 ) }{\varepsilon \,\omega }}, \end{aligned}$$

where $$G=(-{{ c_{9}}}\varepsilon + 2\,\omega \,{ c_{18}}+\varepsilon \,{ c_{17}} ) { c_{9}}-2\,{ c_{4}} \,\omega \,\varepsilon \,{ c_{18}}$$, $$K= (2\,{{ c_{9}}}\omega + -2{ c_{4}}\,\omega \,\varepsilon +{c_{18}} \varepsilon -2{ c_{17}}\,\omega ) { c_{9}}+2{ c_{17}}\,{ c_{4}}\,\omega \,\varepsilon$$.

*Case* 3 $$\alpha \ne 0, \ \beta =0.$$

For a special case when $\alpha \ne 0, \ \beta =0$, One stable approximate first integral $I_{20}$ is derived in addition to the previously obtained unstable approximate first integrals. $I_{9},\ I_{10},\ I_{11},\ I_{12},\ I_{13},\ I_{14},\ I_{15}$.

$$\begin{aligned} I_{20}&=-p_x p_y-\omega ^2 x y-\frac{1}{2}\varepsilon \Bigg [(x-\frac{1}{3}\alpha x^3) p_x-(-\alpha x^2 y+y)p_y\Bigg ]. \end{aligned}$$

Utilizing different combinations of approximate first integrals we obtained the following solutions of this special type of VdP equation. For the first solution we combine $I_{9},\ I_{14}, I_{15},\ I_{20}$ and the second solution is obtained by a combination of $I_{9},\ I_{14}, I_{15}$.

22 $$\begin{aligned} x(t)&= {\frac{\sqrt{\alpha }(\sin (\omega \,t)-\cos ( \omega \,t )) { c_{20}}+(\cos (\omega \,t)-\sin ( \omega \,t )) { c_{15}}}{\omega }},\nonumber \\ y ( t )&={\frac{-\cos ( \omega \,t ) { c_{14}}+\sin ( \omega \,t ) { c_{15}}}{\omega \,\varepsilon }}, \end{aligned}$$

23 $$\begin{aligned} x(t)&={\frac{ 2\,( { c_{20}}\,\varepsilon -{ c_{9}} ) ( \cos ( \omega \,t ) { c_{15}}+\sin ( \omega \,t ) { c_{14}} ) }{\varepsilon \, ( -\cos ( \omega \,t ) { c_{14}}+\sin ( \omega \,t ) { c_{15}} ) ^{2}}},\nonumber \\ y ( t )&={\frac{-\cos ( \omega \,t ) { c_{14}}+\sin ( \omega \,t ) { c_{15}}}{\omega \,\varepsilon }}, \end{aligned}$$

24 $$\begin{aligned} x ( t )&={\frac{{ c_{9}}}{-\sin ( \omega \,t ) \omega \,{ c_{15}}+{ c_{14}}\,\cos ( \omega \,t ) \omega }},\quad y( t ) ={\frac{-\cos ( \omega \,t ) { c_{14}}+\sin ( \omega \,t ) { c_{15}}}{\omega \,\varepsilon }}. \end{aligned}$$

It is important to realize that when we choose $\beta =0$, the Eq. (2) gives the asymptotic solution $x(t)= 2\sqrt{\alpha } \cos (\omega t)$ as $\varepsilon \rightarrow 0$. And the same behavior of this special type of oscillator is obtained in Eq. (22) for specific values of the constants. Also, the equation in *y* is unidirectionally coupled, so we can ignore the dynamics of *y* as it has no effect on the original equation.

*Case* 4 $$\alpha \ne 0, \ \beta \ne 0.$$

Now we discuss a broad class of van der pol equation with arbitrary $\alpha$ and $\beta$. After some lengthy algebraic manipulations, we derive the seven unstable approximate first integrals $I_{9},\ I_{10},\ I_{11},\ I_{12},\ I_{13},\ I_{14},\ I_{15}$ listed in previous cases and one new stable approximate first integral

$$\begin{aligned} I_{21}&=-p_x p_y-\omega ^2 x y-\frac{1}{2}\varepsilon \Bigg [(-\beta x y^2+x-\frac{1}{3}\alpha x^3)p_x-(-\alpha y x^2+y-\frac{1}{3}\beta y^3)p_y\Bigg ]. \end{aligned}$$

Corresponding to these approximate first integrals, we obtain the following solutions of the Eq. (2)

25 $$\begin{aligned} x( t )&=\frac{1}{\sqrt{\alpha }}+{\frac{\sqrt{ \sin ( \omega \,t ) \beta \, c_{15}(2\,\cos ( \omega \,t )\, c_{14}- \sin ( \omega \,t ){ c_{15}})-\cos ^2( \omega \,t )\beta \,{ c_{14}}^{2} }}{\sqrt{3\,\alpha \,\varepsilon ^2 \,\omega ^2}}},\nonumber \\ y( t )&={\frac{-\cos ( \omega \,t ) c_{14}+\sin ( \omega \,t ) c_{15}}{ \omega \,\varepsilon }},\nonumber \end{aligned}$$

26 $$\begin{aligned} x( t )&={\frac{\cos ( \omega \,t ) ( c_{9}\, c_{14}+2\, c_{10}\, c_{15}\,\omega )+\sin ( \omega \,t )(2\, c_{10}\, c_{14}\,\omega - c_{9}\, c_{15})}{{ c_{15}}^{2}\omega +{ c_{14}}^{2}\omega }}, \\ y( t )&={\frac{-\cos ( \omega \,t ) c_{14}+\sin ( \omega \,t ) c_{15}}{ \omega \,\varepsilon }},\nonumber \end{aligned}$$

27 $$\begin{aligned} x( t )&={\frac{ c_{11}}{\cos ( \omega \,t ) c_{15}-\sin ( \omega \,t ) c_{14}}},\quad y( t ) ={\frac{-\cos ( \omega \,t ) c_{14}+\sin ( \omega \,t ) c_{15}}{ \omega \,\varepsilon }}. \end{aligned}$$

### Applications

We have calculated the exact solutions for a wide range of Van der Pol equation. These solutions are described in the form of different cases, and each case belongs to a different type of VdP equation. Here, we discuss some examples of such oscillators and present comparisons of ADHM with numerical solutions.

#### Example 1

Consider a Van der Pol equation with initial value condition

$$\begin{aligned} \ddot{x}-\varepsilon {\dot{x}}+\omega ^2 x=0,\quad x(0)=a,\quad {\dot{x}}(0)=0. \end{aligned}$$

Comparing with the standard Van der Pol Eq. (2), we have $\alpha =0,\,\beta =0.$ Closed-form solutions of generalized VdP equation are discussed in detail in "Closed form solutions of van der pol oscillator" section. For this special type the closed-form solutions are presented in Eqs. (13)–(18). We apply the given conditions to Eqs. (13), (14), (15) and called them ADHM 1, ADHM 2, ADHM 3 foe further correspondence.

**Figure 1:**
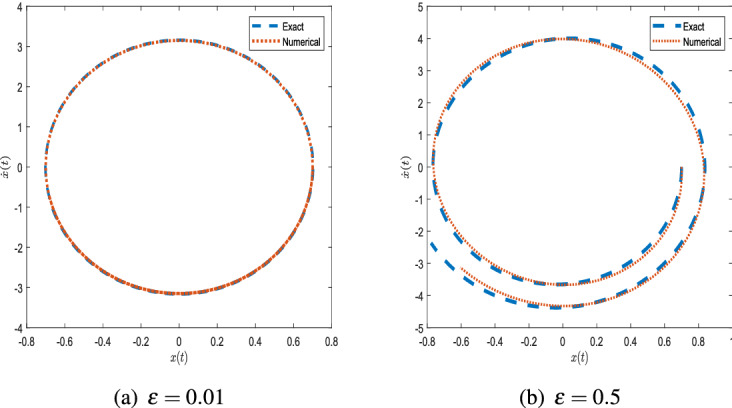
Phase plots with $x_0 = 0.7$ and $\omega = 4$.

Figure 1 shows the phase space diagram generated using the ADHM for $\varepsilon =0.01$ and $\varepsilon =0.5$ compared with the numerical results obtained by using MATLAB ode45 function. It can be observed from the graph that both results are in good agreement.

**Figure 2:**
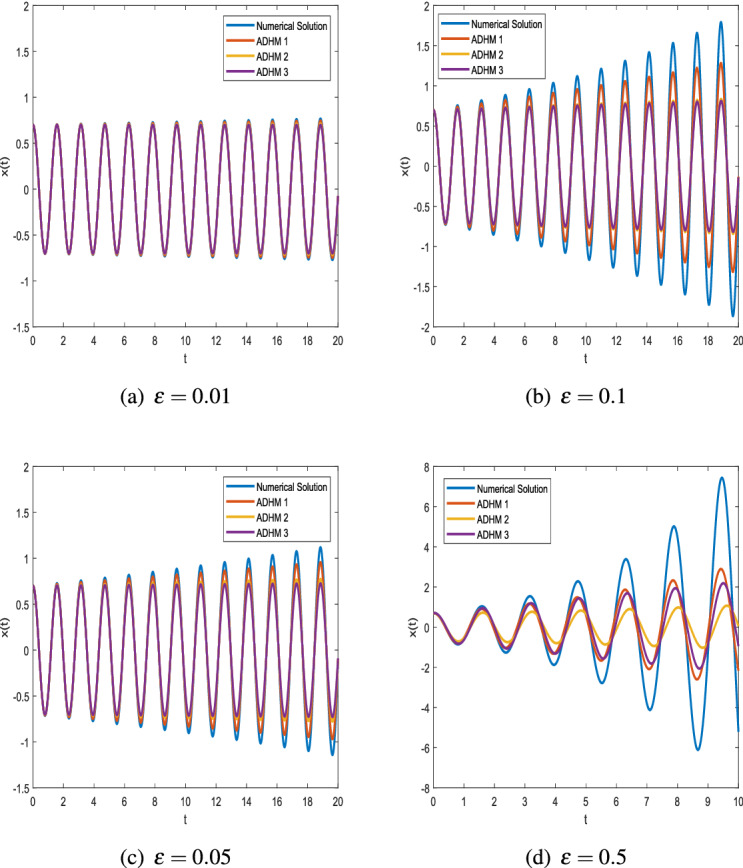
Comparison of ADHM results and numerical solution for parametric values $x_0 = 0.7$ and $\omega = 4$.

**Figure 3:**
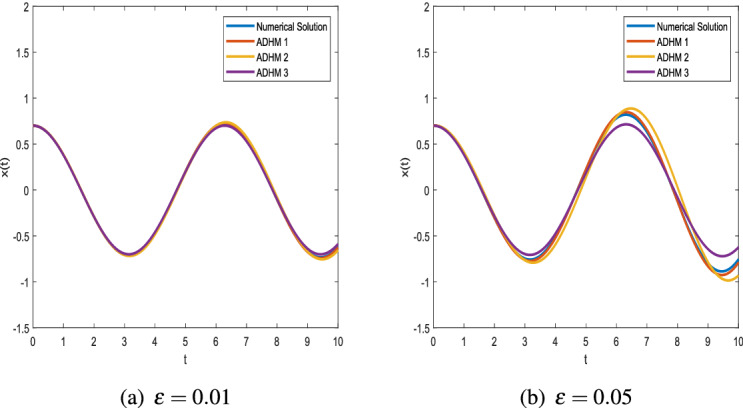
Comparison of ADHM results and numerical solution for parametric values $x_0 = 0.7$ and $\omega = 1$.

**Figure 4:**
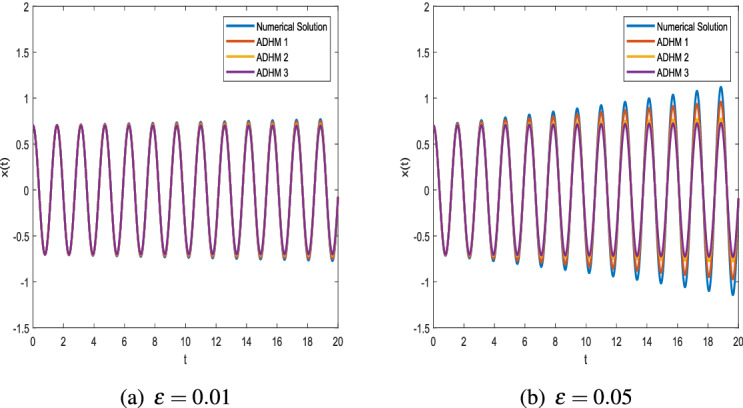
Comparison of ADHM results and numerical solution for parametric values $x_0 = 0.5$ and $\omega = 4$.

Figure 2 shows that there is a minimal difference between the results of ADHM solution and numerical solution. We present the graphs of ADHM solution at $\omega = 4$ and $x_0=0.7$ when $\varepsilon =0.01,\ \varepsilon =0.1,\ \varepsilon =0.05,\ \varepsilon =0.5$. The results are compared with the numerical results for the same parametric values. The insignificant difference between the results motivates us to approximate results for different initial values, and in Fig. 3 we compare the results for $\varepsilon =0.01,\ \varepsilon =0.05$ when $\omega =1$. In Fig. 4 the comparison graphs for two different values of $\varepsilon$ is obtained for fixed values of $\omega$.

#### Example 2

In Example 2, the VdP equation with initial boundary conditions is solved by directly applying the results of ADHM and comparisons between the numerical and exact solutions are presented here after.

$$\begin{aligned} \ddot{x}+\varepsilon (\alpha \ x^2-1){\dot{x}}+\omega ^2 x=0,\quad x(0)=x_0,\quad {\dot{x}}(0)=0. \end{aligned}$$

Comparing with the standard Van der Pol Eq. (2), we have $\alpha \ne 0,\,\beta =0.$

**Figure 5:**
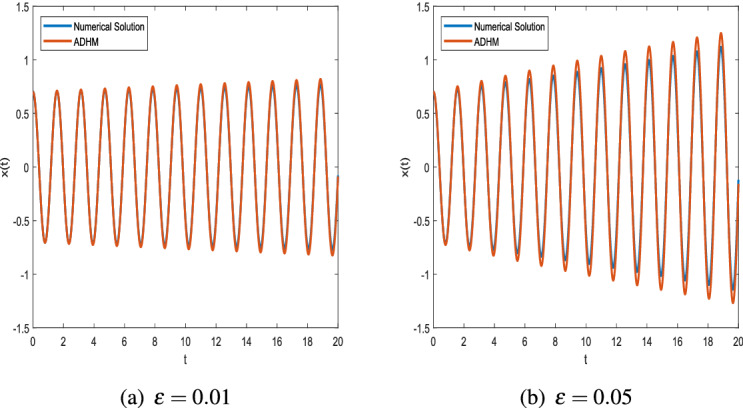
Comparison of ADHM results and numerical solution for parametric values $x_0 = 0.7$ and $\omega = 4$.

**Figure 6:**
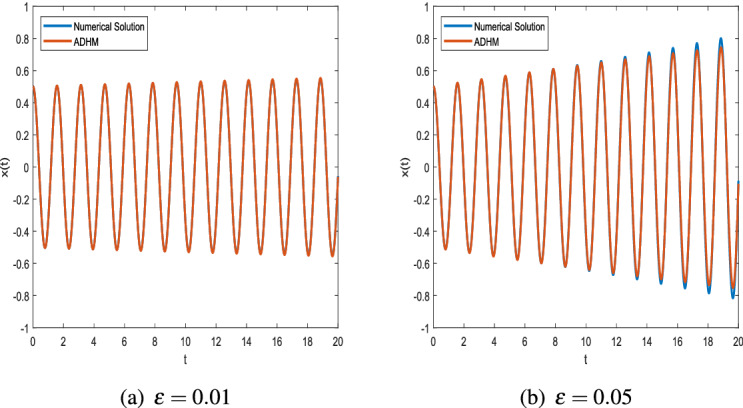
Comparison of ADHM results and numerical solution for parametric values $x_0 = 0.5$ and $\omega = 4$.

Figure 5 shows the effect of increasing the parameter on the accuracy of ADHM. For different values of epsilon, further comparison between the closed closed-form solution and numerical solution with given initial value and ω=1 is made in Fig. 6.

It can be seen from the examples considered in this paper that the ADHM well handle the dissipative system.

## Forced van der pol oscillator

Consider a Forced Van der Pol oscillator

28 $$\begin{aligned} \ddot{x}+\varepsilon (\alpha x^2+\beta y^2-1){\dot{x}}+(\omega ^2 +F_1 cos(\Omega t))x=0. \end{aligned}$$

First we need to construct an auxiliary equation corresponding to the forced VdPO equation.

29 $$\begin{aligned} \ddot{y}-\varepsilon (\alpha x^2+\beta y^2-1){\dot{y}}+(\omega ^2 +F_1 \cos (\Omega t))y=0. \end{aligned}$$

Since the total system becomes conservative, the Hamiltonian of the system can easily be obtained which satisfies both Eqs. (28) and (29).

30 $$\begin{aligned} H_{fv}=H+xyF_1 \cos (\Omega t). \end{aligned}$$

The next step in the approximate partial Hamiltonian approach is to rewrite the approximate Noether symmetry determining equation listed in Appendix (44) with the approximate dual Hamiltonian (30). However, we are interested in only zeroth-order and first-order approximation and ignore all the higher-order approximations. To obtain the zeroth-order and first-order approximation systems, we separate the determining equation with respect to the powers of $\varepsilon$. The zeroth-order approximation and first-order approximation are given by an over-determined system of differential equations given in B

After some algebraic manipulations, we derive the following first integrals for the forced Van der Pol oscillator.

31 $$\begin{aligned} I_{f1}&=-p_x p_y-\omega ^2 x y-\frac{1}{2}\varepsilon \Bigg [(-\beta x y^2+x-\frac{1}{3}\alpha x^3)p_x-(-\alpha y x^2+y-\frac{1}{3}\beta y^3)p_y\Bigg ], \end{aligned}$$

32 $$\begin{aligned} I_{f2}&=\varepsilon \Bigg [ p_y+\omega ^2 x y\Bigg ]+O(\varepsilon ),\end{aligned}$$

33 $$\begin{aligned} I_{f3}=&\, \varepsilon \Bigg [-2 \Bigg (p_y+\omega ^2 x y\Bigg ) \int \mathbf{Se \Bigg (\frac{16 \omega ^2}{\Omega ^2}\,,\,-\frac{8F_1}{\Omega ^2}~,~\frac{1}{2}~\Omega t\Bigg )\mathbf{dt} }+(p_x ~x+p_y~y)\nonumber \\&\mathbf{Se} \Bigg (\frac{16 \omega ^2}{\Omega ^2}\,,\,-\frac{8F_1}{\Omega ^2}~,~\frac{1}{2}~\Omega t\Bigg ) - \frac{1}{2} x y~\Omega ~ \mathbf{Se }^\prime \Bigg (\frac{16 \omega ^2}{\Omega ^2}\,,\,-\frac{8F_1}{\Omega ^2}~,~\frac{1}{2}~\Omega t\Bigg )\Bigg ],\end{aligned}$$

34 $$\begin{aligned} I_{f4}=&\,\varepsilon \Bigg [-2 \Bigg (p_y+\omega ^2 x y\Bigg ) \int \mathbf{Ce \Bigg (\frac{16 \omega ^2}{\Omega ^2}\,,\,-\frac{8F_1}{\Omega ^2}~,~\frac{1}{2}~\Omega t\Bigg )\mathbf{dt} }+(p_x ~x+p_y~y)\nonumber \\&\mathbf{Ce} \Bigg (\frac{16 \omega ^2}{\Omega ^2}\,,\,-\frac{8F_1}{\Omega ^2}~,~\frac{1}{2}~\Omega t\Bigg ) - \frac{1}{2} x y~\Omega ~ \mathbf{Ce }^\prime \Bigg (\frac{16 \omega ^2}{\Omega ^2}\,,\,-\frac{8F_1}{\Omega ^2}~,~\frac{1}{2}~\Omega t\Bigg )\Bigg ],\end{aligned}$$

35 $$\begin{aligned} I_{f5}&=\varepsilon p_x \mathbf{Ce} \Bigg (\frac{4 \omega ^2}{\Omega ^2}\,,\,-\frac{2F_1}{\Omega ^2}~,~\frac{1}{2}~\Omega t\Bigg )-\frac{1}{2}\varepsilon y~\Omega ~\mathbf{Ce} ^\prime \Bigg (\frac{4 \omega ^2}{\Omega ^2}\,,\,-\frac{2F_1}{\Omega ^2}~,~\frac{1}{2}~\Omega t\Bigg ),\end{aligned}$$

36 $$\begin{aligned} I_{f6}&=\varepsilon p_x \mathbf{Se} \Bigg (\frac{4 \omega ^2}{\Omega ^2}\,,\,-\frac{2F_1}{\Omega ^2}~,~\frac{1}{2}~\Omega t\Bigg )-\frac{1}{2}\varepsilon y~\Omega ~\mathbf{Se} ^\prime \Bigg (\frac{4 \omega ^2}{\Omega ^2}\,,\,-\frac{2F_1}{\Omega ^2}~,~\frac{1}{2}~\Omega t\Bigg ), \end{aligned}$$

where $\mathbf{Ce} ,\,\mathbf{Se} ,\, \mathbf{Ce} ^\prime ,\, \mathbf{Se} ^\prime$ denotes even *Mathieu* function, odd *Mathieu* function, derivative of even *Mathieu* function and derivative of odd *Mathieu* function respectively.

## Liénard system

Consider a more general type of linear second order differential equation called liénard equation

37 $$\begin{aligned} \ddot{x}+\varepsilon f(x) {\dot{x}} +\omega ^2 x=0, \end{aligned}$$

where *f*(*x*) is a differentiable function. Our strategy is, to introduce a two dimensional autonomous auxiliary dynamical system in such a way that a Hamiltonian cab be written for the system of equation. We consider the following auxiliary equation

38 $$\begin{aligned} \ddot{y}-\varepsilon f(x) {\dot{y}} +\omega ^2 y=0. \end{aligned}$$

Now by trivial procedure, we can write the Lagrangian

39 $$\begin{aligned} L={\dot{x}}{\dot{y}}-\omega ^2 x y-\frac{\varepsilon }{2}\bigg [f(x) y {\dot{x}}-\int {f(x)}dx ~{\dot{y}}\bigg ], \end{aligned}$$

for (37) and corresponding auxiliary Eq. (38). It is worthy to mention here that a Lagrangian is not unique and one can use different Lagrangians. The corresponding Hamiltonian is given by

40 $$\begin{aligned} H=p_x p_y+\omega ^2 x y +\frac{\varepsilon }{2}\bigg [f(x) y p_y -\int {f(x)}dx ~p_x -\frac{\varepsilon }{2} f(x) y~\int {f(x)}dx \bigg ], \end{aligned}$$

with conjugate momenta $p_x={\dot{y}}-\frac{\varepsilon }{2} f(x) y$ and $p_y={\dot{x}}+\frac{\varepsilon }{2} \int {f(x)} dx$.

Thus in accordance with the partial Hamiltonian approach the Liénard equation with auxiliary equation is solved for all possible first integrals. After some lengthy calculations as discussed in previous sections we calculated the generalized first integral for two different cases.

*Case* 1 $$\frac{d~f(x)}{dx}=0$$, $$f(x)=f$$(constant).

In this case, we find

41 $$\begin{aligned} \phi _{1}&=-p_x p_y-\omega ^2 x y-\frac{1}{2}\varepsilon \Bigg [x p_x f-y p_y f\Bigg ],\nonumber \\ \phi _{2}&=p_x\sin (\omega t)- \omega y \cos (\omega t)-\frac{1}{2}\varepsilon f\Bigg [(\omega ^2 t y-p_x)\frac{\cos (\omega t)}{ \omega }-(p_x t +y)\sin (\omega t)\Bigg ], \nonumber \\ \phi _{3}&=p_x\cos (\omega t)+ \omega y \sin (\omega t)+\frac{1}{2}\varepsilon f\Bigg [p_x~t\cos (\omega t)+\omega t y\sin (\omega t)\Bigg ], \nonumber \\ \phi _{4}&=-\varepsilon \Bigg [p_x p_y+\omega ^2 x y\Bigg ]+O(\varepsilon ^2),\nonumber \\ \phi _{5}&=\varepsilon \Bigg [\frac{1}{2}p_x x-\frac{1}{2} p_y y\Bigg ],\nonumber \\ \phi _{6}&=\varepsilon \Bigg [\cos (2 \omega t)(p_x x+p_y y)+(\omega ^2 xy-p_x p_y) \frac{sin(2 \omega t)}{\omega }\Bigg ]+O(\varepsilon ^2),\nonumber \\ \phi _{7}&=\varepsilon \Bigg [(x p_x+y p_y)\sin (2\omega t)+\frac{ \cos (2\omega t)}{\omega }( \omega ^2 xy-p_x p_y)\Bigg ]+O(\varepsilon ^2), \nonumber \\ \phi _{8}&=-2\varepsilon \Bigg [p_x p_y+\omega ^2 x y\Bigg ]+O(\varepsilon ^2), \nonumber \\ \phi _{9}&=\varepsilon \bigg [p_x \sin (\omega t)-\omega y \cos (\omega t)\Bigg ], \nonumber \\ \phi _{10}&=\varepsilon \bigg [p_x \cos (\omega t)+\omega y \sin (\omega t)\Bigg ]. \end{aligned}$$

For $f=-1$ the Liénard equation reduced to

$$\begin{aligned} \ddot{x}-\varepsilon {\dot{x}}+\omega ^2 x=0, \end{aligned}$$

which is a particular form of VDPO with $\alpha = 0$ and $\beta = 0$. By comparing the results with the previously obtained results for this specific case, we found the same first integral in each calculation. These observations strengthen our claim for finding a general form of the first integral, which saves the time and effort of lengthy calculations.

*Case* 2 When $$\frac{d~f(x)}{dx} \ne 0$$.

The routine calculations leads to

42 $$\begin{aligned} \phi _{11}&=-p_x p_y-\omega ^2 x y-\frac{1}{2}\varepsilon \Bigg [-\int {f(x)}dx p_x+f(x) y p_y\Bigg ],\nonumber \\ \phi _{12}&=-\varepsilon \Bigg [p_x p_y+\omega ^2 x y\Bigg ]+O(\varepsilon ^2), \nonumber \\ \phi _{13}&=\varepsilon \frac{1}{2}\Bigg [-p_x x+ p_y y\Bigg ], \nonumber \\ \phi _{14}&=\varepsilon \Bigg [(x p_x +y p_y)\cos (2 \omega t)- (-\omega ^2 xy+p_x p_y)\frac{\sin (2 \omega t)}{\omega }\Bigg ]+O(\varepsilon ^2), \nonumber \\ \phi _{15}&=\varepsilon \Bigg [(x p_x+y p_y)\sin (2\omega t)-(- \omega ^2 xy+p_x p_y)\frac{ \cos (2\omega t)}{\omega }\Bigg ]+O(\varepsilon ^2),\nonumber \\ \phi _{16}&=-2\varepsilon \Bigg [p_x p_y+\omega ^2 x y\Bigg ]+O(\varepsilon ^2), \nonumber \\ \phi _{17}&=\varepsilon \Bigg [p_x \sin (\omega t)-\cos (\omega t)\omega y\Bigg ],\nonumber \\ \phi _{18}&=\varepsilon \Bigg [p_x \cos (\omega t)+\sin (\omega t)\omega y\Bigg ]. \end{aligned}$$

We take different forms of *f*(*x*) and determine the first integrals for the subsequent systems. Therefore, for this we proceed as:

Let $f(x)=\alpha x^2 -1$, then the Liénard equation takes the form

$$\begin{aligned} \ddot{x}+\varepsilon (\alpha x^2 -1) {\dot{x}} +\omega ^2 x=0. \end{aligned}$$

We can observe that it is a VdP equation with $\beta =0$ and all the first integrals are the same as discussed in the previous section. Thus, we have found that our method can be applied to a wide range of non-conservative systems.

## Conclusions

We have presented a novel approach of the approximate Hamiltonian method to find the solutions of dissipative system. The approximate Hamiltonian method is only applicable to the conservative system. The approach presented in this article is applicable to both conservative and non-conservative systems. We were able to obtain an approximate dual Hamiltonian for a non-conservative system, which leads further to the construction of the first integrals and closed-form solutions. It is worth mentioning that we can choose any arbitrary initial condition for the auxiliary system as we are not interested in its dynamics. Our adaptation of approximate Hamiltonian for non-conservative systems and its application in deriving the closed-form solution is practical.

In this paper, we established approximate dual Hamiltonian for non-conservative systems. Then by utilizing the approximate Hamiltonian approach, we derived the first integrals for the system under certain parametric conditions. Finally, using the approximate first integrals, we obtained exact solutions of the VdP equation. For more insight, the ADHM is utilized to compute the closed-form solutions of the Van der Pol initial value problems with different initial conditions. The numerical results are developed by using MATLAB ode45 and presented graphically. From illustrative examples, it is found that the exact solutions obtained by using ADHM are in good agreement with the numerical solutions.

## A Approximate hamiltonian version of approximate Noether theorem

### Definition 1

Let $L_0+\varepsilon \, L_1$ be an approximate Lagrangian and $H_0+\varepsilon \, H_1$ be the corresponding approximate Hamiltonian is connected by the Legendre transformation

43 $$\begin{aligned} H_0+\varepsilon \, H=p^i \,{\dot{q}}^i-L-\varepsilon \, L \end{aligned}$$

and satisfies the following first order approximate Hamiltonian system

$$\begin{aligned} {\dot{q}}^i&=\frac{\partial H}{\partial p^i}+O(\varepsilon ^2),\\ {\dot{p}}^i&=-\frac{\partial H}{\partial p^i}+O(\varepsilon ^2). \end{aligned}$$

### Definition 2

The approximate operator

$$\begin{aligned} X=X_0+\varepsilon X_1 \end{aligned}$$

is known as the approximate symmetry generator corresponding to the approximate Hamiltonian equation if it admits the approximate Hamiltonian determining equation

44 $$\begin{aligned} \zeta _t\, \frac{\partial H}{\partial p} + p D_t(\eta )-X(H)-H\,D_t(\xi )-D_t(B)=0, \end{aligned}$$

where $$\xi =\xi _0+\varepsilon \xi _1$$, $$\eta =\eta _0+\varepsilon \eta _1$$ and $$B=B_0+\varepsilon B_1$$.

### Definition 3

Approximate first integral of the system is defined as

45 $$\begin{aligned} I(t,q_i,{\dot{q}}_i;\varepsilon )=I_0(t,q_i,{\dot{q}}_i;\varepsilon )+\varepsilon \,I_1(t,q_i,{\dot{q}}_i;\varepsilon ) \end{aligned}$$

for every solution of the system if $D_t(I_0+\varepsilon I_1)=O(\varepsilon ^2)$.

## B The expression for the zeroth and first approximation of forced van der pol equation

Zeroth order approximation:

46 $$\begin{aligned}{}&\xi _{0x}=0,\nonumber \\&\xi _{0y}=0,\nonumber \\&\eta ^2_{0x}=0,\nonumber \\&\eta ^1_{0y}=0,\nonumber \\&\eta ^2_{0t}-B_{0x}=0,\nonumber \\&\eta ^1_{0t}-B_{0y}=0,\nonumber \\&\eta ^1_{0x}+\eta ^2_{0y}-\xi _{0t}=0,\nonumber \\&(\omega ^2+F_1 \cos (\Omega t))(\xi _{0t} x y+\eta ^1_{0} y +\eta ^2_{0} x) +B_{0t}=0. \end{aligned}$$

First order approximation:

47 $$\begin{aligned}{}&\xi _{1y}=0,\nonumber \\&\xi _{1x}=0,\nonumber \\&\eta ^1_{1y}=0,\nonumber \\&\eta ^2_{1x}=0,\nonumber \\&\xi _{1t}+\eta ^2_{1y}-\eta ^1_{1x}=0,\nonumber \\&\eta ^2_{0} \beta x y-B_{1y}+\frac{1}{2}\eta ^1_{0}( \alpha x^2+\beta y^2 - 1 ) - \frac{1}{2} \eta ^2_{0y}(x-\beta x y^2 -\frac{1}{3}\alpha x^3)+\eta ^1_{1t}=0,\nonumber \\&\frac{1}{2}\eta ^2_{0}(\alpha x^2+\beta y^2 - {1}) +\frac{1}{2}\eta ^1_{0x}(\alpha x^2 y + \frac{1}{3} \beta y^3 - y)+\eta ^1_0 \alpha x y - \eta ^2_{1t} + B_{1x}=0,\nonumber \\&\frac{1}{2}B_{0y}(\alpha y x^2 +\frac{1}{3}\beta y^3-y)-(\xi _{1t} x y+\eta ^1_{1} y+\eta ^2_{1} x)(\omega ^2+F_1 \cos (\Omega t))+B_{1t}\nonumber \\&+\frac{1}{2} B_{0x}(x-\beta x y^2 -\frac{1}{3}\alpha x^3)=0. \end{aligned}$$

.
